# EPA-Derived diHEPAs Attenuate Lipopolysaccharide-Induced Acute Lung Injury by Regulating Inflammation and Redox Homeostasis

**DOI:** 10.3390/ijms27083373

**Published:** 2026-04-09

**Authors:** Yan Su, Soon Kyu Kwon, Hack Sun Choi, Yunjon Han, Jung-Hee Park, Yong-Suk Jang, Jong Hyun Choi, Jeong-Woo Seo

**Affiliations:** 1 Microbial Biotechnology Research Center, Korea Research Institute of Bioscience and Biotechnology (KRIBB), Jeongeup-si 56212, Republic of Korea; suyan@kribb.re.kr (Y.S.); ksk0469@kribb.re.kr (S.K.K.); gugin@kribb.re.kr (Y.H.); 2 Department of Biotechnology, Jeonbuk National University, Iksan 54596, Republic of Korea; junghee.park@jbnu.ac.kr; 3 Department of Biochemistry & Molecular Biology, Yonsei University College of Medicine, Seoul 03722, Republic of Korea; choix074@yuhs.ac; 4 Department of Molecular Biology, Jeonbuk National University, Jeonju 54896, Republic of Korea; yongsuk@jbnu.ac.kr; 5 Department of Bioactive Material Sciences, Jeonbuk National University, Jeonju 54896, Republic of Korea

**Keywords:** acute lung injury, EPA-derived lipid mediators, inflammation resolution, oxidative stress

## Abstract

Acute lung injury (ALI) is characterized by excessive inflammation, oxidative stress, and impaired resolution responses, partly driven by dysregulated macrophage activation. In this study, a defined mixture of eicosapentaenoic acid (EPA)-derived dihydroxyeicosapentaenoic acids (diHEPAs), comprising 5,15-diHEPA and 8,15-diHEPA at an equimolar ratio, was generated using soybean lipoxygenase and its protective effects on lipopolysaccharide (LPS)-induced ALI were investigated. Mice were orally administered 5,15-diHEPA (40 μg/kg), 8,15-diHEPA (40 μg/kg), or the diHEPA mixture (20 μg/kg each) for 7 days before LPS challenge. LPS exposure induced severe lung injury, as evidenced by an increased lung wet/dry ratio, inflammatory cell infiltration, and oxidative stress. Treatment with diHEPAs attenuated lung pathological damage, reduced proinflammatory cytokine production, and restored redox homeostasis. Consistently, in vitro studies in RAW264.7 macrophages showed that the diHEPA mixture suppressed LPS-induced inflammatory responses through the inhibition of NF-κB signaling and rebalanced oxidative stress via modulation of the NOX2/Nrf2/HO-1/ROS axis. Altogether, these results indicate that EPA-derived diHEPAs confer protection against ALI by suppressing inflammation and restoring redox balance, emphasizing their potential as therapeutic agents for ALI.

## 1. Introduction

Acute lung injury (ALI) and its severe form, acute respiratory distress syndrome (ARDS), are life-threatening clinical conditions characterized by diffuse alveolar damage, excessive inflammatory responses, and profound oxidative stress [1,2]. The pathogenesis of ALI involves a tightly coordinated yet frequently dysregulated immune response in which uncontrolled inflammation and redox imbalance synergistically drive epithelial and endothelial barrier disruption, pulmonary edema, and respiratory failures [3]. Despite advances in supportive care, effective pharmacological therapies targeting the underlying mechanisms underlying ALI remain limited.

Lipopolysaccharide (LPS)-induced ALI is a widely used experimental model that recapitulates important pathological features of human ALI, including neutrophil infiltration, macrophage activation, cytokine overproduction, and disruption of alveolar–capillary barrier integrity [4,5,6,7]. Following LPS exposure, innate immune cells, particularly neutrophils and macrophages, are rapidly activated and exhibit a proinflammatory phenotype, producing high levels of tumor necrosis factor-α (TNF-α), interleukins (ILs), and reactive oxygen species (ROS), which further exacerbate lung injury [8].

A vital pathogenic feature of ALI is excessive oxidative stress resulting from uncontrolled ROS production in the inflamed lung [9,10]. Under physiological conditions, ROS act as essential signaling molecules in host defense; however, during ALI, sustained ROS production overwhelms endogenous antioxidant systems, leading to lipid peroxidation, protein oxidation, DNA damage, and the disruption of alveolar–capillary integrity [11,12,13]. Among the major sources of ROS in ALI, NADPH oxidase 2 (NOX2) plays a pivotal role [14,15]. NOX2 is highly expressed in infiltrating neutrophils and macrophages and is rapidly activated by inflammatory stimuli, including LPS and cytokines [16,17].

To counteract oxidative injury, cells depend on endogenous antioxidant defense systems primarily regulated by nuclear factor erythroid 2-related factor 2 (Nrf2). Upon activation, Nrf2 translocates to the nucleus and induces the expression of a wide array of cytoprotective genes, including heme oxygenase-1 (HO-1), superoxide dismutase, catalase, and glutathione-related enzymes, thereby restricting ROS accumulation and restoring redox homeostasis [18,19,20,21]. Impaired Nrf2 signaling is associated with exaggerated inflammation, sustained oxidative stress, and delayed resolution in ALI, underscoring its essential role in maintaining lung integrity and immune homeostasis [22,23].

Specialized pro-resolving mediators (SPMs) derived from omega-3 polyunsaturated fatty acids, particularly eicosapentaenoic acid (EPA) and docosahexaenoic acid (DHA), play pivotal roles in the inflammation resolution processes [24,25,26,27]. EPA-derived SPMs, such as resolvin E1 and resolvin E2, exert potent pro-resolving effects through the engagement of specific G protein-coupled receptors, including ChemR23 and BLT1, resulting in macrophage reprogramming toward a pro-resolving phenotype [28,29,30]. Dihydroxyeicosapentaenoic acids (diHEPAs), including 5,15-diHEPA and 8,15-diHEPA, are EPA-derived lipid mediators generated through lipoxygenase-dependent pathways and are increasingly recognized as SPM-like molecules with anti-inflammatory and cytoprotective properties [31,32]. In this study, we generated a defined diHEPA mixture using soybean lipoxygenase, consisting of 5,15-diHEPA and 8,15-diHEPA (1:1). However, there is no clear information on the potential role of this defined EPA-derived diHEPA mixture in regulating macrophage-driven inflammation, oxidative stress, and lung injury resolution in ALI. Therefore, we aimed to test the hypothesis that the diHEPA mixture confers protection against LPS-induced ALI by reprogramming macrophage inflammatory responses and restoring redox homeostasis, using an LPS-induced ALI mouse model and RAW264.7 macrophages in vitro.

## 2. Results

### 2.1. diHEPAs Attenuated LPS-Induced Lung Injury

**Figure 1:**
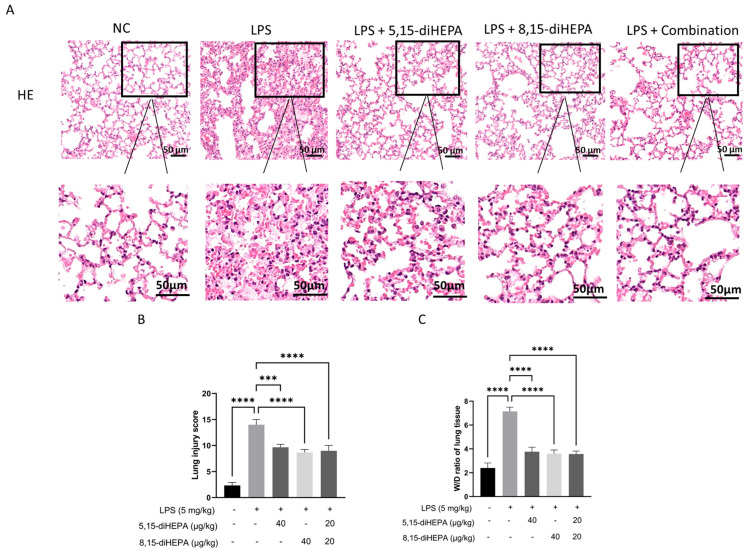
diHEPAs attenuated LPS-induced acute lung injury in mice. (**A**) Representative hematoxylin and eosin (H&E)-stained lung sections from the control, LPS (5 mg/kg), LPS + 5,15-diHEPA (40 μg/kg), LPS + 8,15-diHEPA (40 μg/kg), and LPS + combination treatment (5,15-diHEPA 20 μg/kg + 8,15-diHEPA 20 μg/kg) groups. Scale bar, 50 μm. (**B**) The injury score. Data are expressed as mean ± SD and analyzed using the Kruskal–Wallis test followed by Dunn’s post hoc test. *** *p* < 0.001, **** *p* < 0.0001. (**C**) The W/D ratio was measured in the LPS-induced ALI model. Data are expressed as mean ± SD and analyzed using Tukey’s test. **** *p* < 0.0001.

To determine the protective effects of EPA-derived diHEPAs on ALI, mice were treated with 5,15-diHEPA (40 μg/kg), 8,15-diHEPA (40 μg/kg), or a combination of both diHEPAs (20 μg/kg each) prior to LPS challenge. Histopathological examination of lung sections revealed that LPS exposure resulted in marked lung injury, including alveolar wall thickening, inflammatory cell infiltration, and alveolar collapse (Figure 1A,B). The lung W/D ratio further indicated that LPS exposure caused edema compared with that in the NC group (*p* < 0.0001; Figure 1C). Treatment with either 5,15-diHEPA or 8,15-diHEPA significantly alleviated these pathological changes.

### 2.2. diHEPAs Reduced Inflammation in Bronchoalveolar Lavage Fluid (BALF)

**Figure 2:**
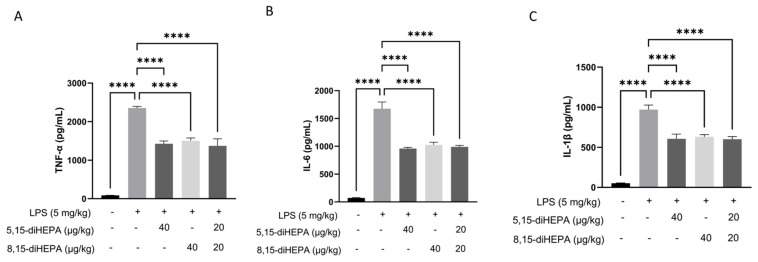
diHEPAs reduced pulmonary inflammation and cellular infiltration in BALF. (**A**–**C**) Concentrations of proinflammatory cytokines (TNF-α, IL-6, and IL-1β) in BALF, determined by ELISA. Data are expressed as mean ± SD and analyzed using Tukey’s test. **** *p* < 0.0001.

LPS exposure significantly increased the levels of TNF-α, IL-6, and IL-1β to 2351.88 ± 42.04, 1674 ± 122.83, and 969.96 ± 58.31 pg/mL, respectively, in BALF (*p* < 0.0001 vs. NC group, each). However, treatment with both diHEPAs effectively moderated these cytokine levels to approximately 60% compared with those in the ALI group (Figure 2A–C).

### 2.3. diHEPAs Suppressed Oxidative Stress in Mice with ALI

**Figure 3:**
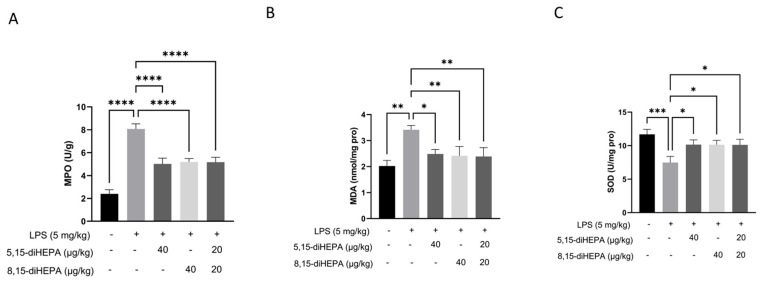
diHEPAs suppressed oxidative stress in ALI mice. (**A**) Myeloperoxidase (MPO) levels in the lung tissue were determined using an ELISA kit. (**B**) Malondialdehyde (MDA) content in lung tissues. (**C**) Activities of superoxide dismutase (SOD) in lung tissues. Data are expressed as mean ± SD and analyzed using Tukey’s test. * *p* < 0.05, ** *p* < 0.01, *** *p* < 0.001, **** *p* < 0.0001.

Myeloperoxidase (MPO) levels were significantly increased in mice with ALI compared with those in mice in the NC group (*p* < 0.0001; Figure 3A). Similarly, malondialdehyde (MDA) levels in mice with ALI were increased significantly compared with those in the NC group (*p* < 0.0001, Figure 3B), along with the reduction in superoxide dismutase (SOD) levels (*p* < 0.0001 each vs. NC group; Figure 3C). However, treatment with diHEPAs decreased the MPO and MDA levels and increased the SOD activity.

### 2.4. diHEPAs Suppressed Proinflammatory Responses In Vitro

**Figure 4:**
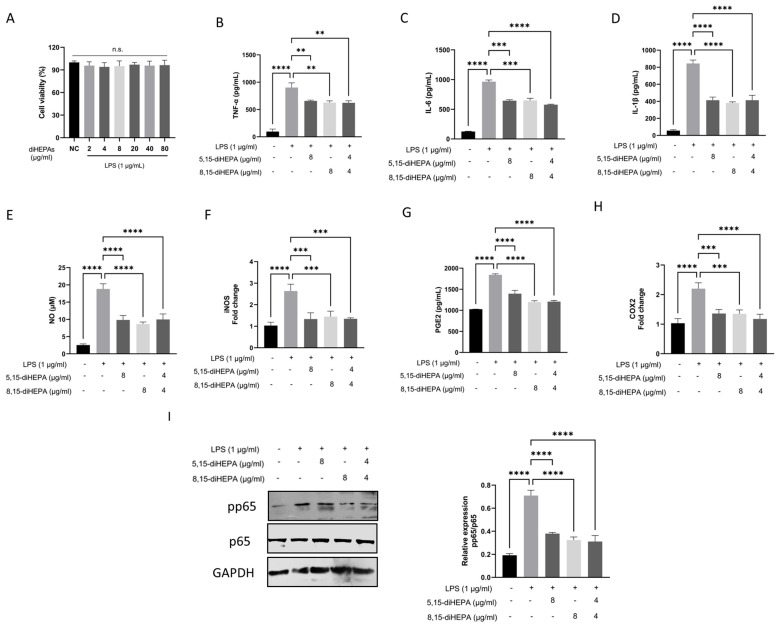
diHEPAs attenuated LPS-induced inflammatory activation in RAW264.7 macrophages. Macrophages were exposed to LPS (1 μg/mL) for 24 h in the presence or absence of 5,15-diHEPA and/or 8,15-diHEPA. (**A**) Cell viability was evaluated using MTT assay. (**B**–**D**) Secretion of the proinflammatory cytokines TNF-α, IL-6, and IL-1β into culture supernatants was determined using ELISA kits. (**E**) Nitric oxide (NO) production was evaluated using the Griess reaction. (**F**) Relative mRNA levels of inducible nitric oxide synthase (iNOS), expressed as fold change compared with that in untreated controls. (**G**) Prostaglandin E2 (PGE2) concentrations in the culture supernatants were determined by ELISA. (**H**) Relative cyclooxygenase-2 (COX-2) expression presented as fold change versus control. (**I**) Phosphorylated p65 (pp65) levels were determined by Western blotting. Data are expressed as mean ± SD and analyzed using Tukey’s test. n.s. no significant difference; ** *p* < 0.01, *** *p* < 0.001, **** *p* < 0.0001.

The MTT assay confirmed that diHEPA concentrations as high as 80 µg/mL combined with LPS (1 μg/mL) were noncytotoxic (Figure 4A). To determine the anti-inflammatory effects of diHEPAs, RAW264.7 macrophages were stimulated with LPS (1 µg/mL) in the presence or absence of 5,15-diHEPA and/or 8,15-diHEPA. As anticipated, LPS robustly induced the secretion of proinflammatory cytokines TNF-α, IL-6, and IL-1β compared with that in unstimulated controls (Figure 4B–D). Treatment with either 5,15-diHEPA (8 µg/mL) or 8,15-diHEPA (8 µg/mL) significantly attenuated this LPS-induced cytokine production. Moreover, cotreatment with both diHEPAs (4 µg/mL each) markedly reduced the levels of TNF-α, IL-6, and IL-1β, indicating a potent inhibitory effect on inflammatory cytokine release.

In parallel, LPS stimulation markedly increased NO production and iNOS expression (Figure 4E,F). However, treatment with either 5,15-diHEPA or 8,15-diHEPA significantly suppressed LPS-induced NO generation and reduced iNOS mRNA levels. The combination treatment also exerted comparable inhibitory effects. Consistent with these findings, LPS exposure strongly increased PGE2 production and COX-2 expression (Figure 4G,H), whereas treatment with either diHEPA derivative significantly decreased PGE2 release and downregulated COX-2 expression compared with LPS alone. The combined treatment with both diHEPAs effectively decreased the LPS-induced PGE2 and COX-2 upregulation.

To further explore the mechanism underlying the anti-inflammatory effects of diHEPAs, we investigated NF-κB signaling by determining the phosphorylation of p65 (pp65) in LPS-stimulated RAW264.7 macrophages (Figure 4I). LPS administration markedly increased the pp65 levels compared with those in the untreated control, confirming robust activation of the NF-κB pathway. However, treatment with either 5,15-diHEPA or 8,15-diHEPA significantly suppressed LPS-induced p65 phosphorylation.

### 2.5. diHEPAs Altered Oxidative Stress In Vitro

**Figure 5:**
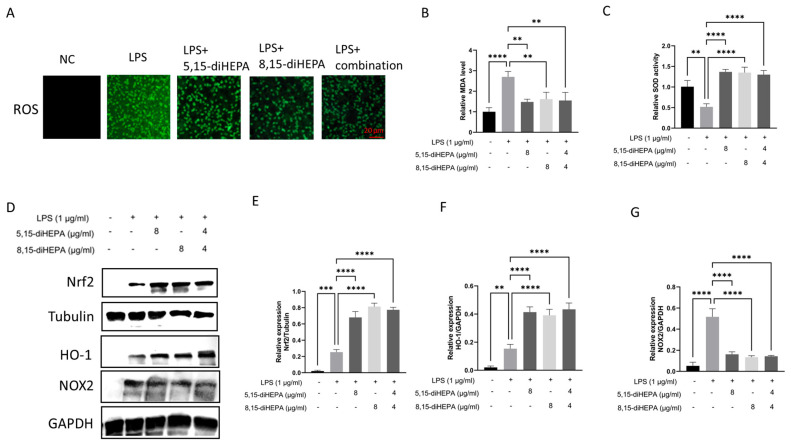
diHEPAs modulated redox balance in RAW264.7 macrophages. (**A**) Representative fluorescence images depicting intracellular reactive oxygen species (ROS) production that was detected using an ROS-sensitive fluorescent probe. Scale bar, 20 μm. (**B**) Malondialdehyde (MDA) levels were quantified and normalized to protein concentration. (**C**) Superoxide dismutase was detected. (**D**–**G**) Representative quantification of NOX2, Nrf2, and HO-1 protein expression determined by Western blotting. Data are expressed as mean ± SD and analyzed using Tukey’s test. ** *p* < 0.01, *** *p* < 0.001, **** *p* < 0.0001.

To determine the antioxidative properties of diHEPAs, oxidative stress markers were examined in LPS-stimulated RAW264.7 macrophages. Intracellular ROS levels were first analyzed using fluorescence staining (Figure 5A), which revealed minimal ROS signals in the negative control, whereas LPS stimulation markedly increased ROS accumulation. Treatment with either 5,15-diHEPA or 8,15-diHEPA substantially reduced LPS-induced ROS generation, while the combined treatment further attenuated ROS levels, indicating a strong antioxidant effect.

LPS exposure significantly decreased SOD activity and increased MDA levels compared with those in controls (Figure 5B,C). Conversely, treatment with both 5,15-diHEPA and 8,15-diHEPA significantly restored SOD activity and reduced MDA accumulation in LPS-treated cells. To further clarify the underlying molecular mechanisms, the NOX2 and Nrf2 antioxidant signaling pathway was examined. LPS exposure modestly induced the expression of NOX2, which was decreased by treatment with diHEPAs (Figure 5D,E). Furthermore, the nuclear activity of Nrf2 and its downstream target HO-1 markedly increased after treatment with 5,15-diHEPA or 8,15-diHEPA (Figure 5D,F,G).

## 3. Discussion

ALI is a severe inflammatory condition characterized by damage to epithelial and endothelial cells, along with disruption of the alveolar–capillary barrier, leading to pulmonary edema and collapse [33]. In the present study, we demonstrated that EPA-derived diHEPAs, specifically 5,15-diHEPA and 8,15-diHEPA (1:1), generated via soybean lipoxygenase, significantly attenuate the pathological features of LPS-induced ALI in both in vivo and in vitro models. Notably, our findings primarily support a preventive role for diHEPAs, as pretreatment markedly reduced the severity of lung injury. Mechanistically, these protective effects are associated with suppression of oxidative stress and modulation of inflammatory responses, consistent with the emerging concept that lipid mediators actively promote the resolution of inflammation rather than simply suppressing it [34,35].

Following LPS challenge, lung tissue exhibited widespread structural disruption, including dense inflammatory cell infiltration, marked thickening of alveolar septa, and expansion of the interstitial space, indicative of severe pulmonary injury. These changes were paralleled by a prominent increase in the lung W/D ratio, indicating excessive fluid accumulation and loss of alveolar–capillary barrier integrity. Conversely, treatment with diHEPAs significantly decreased the W/D ratio, indicating reduced pulmonary edema. Histological examination also revealed preservation of the alveolar architecture, with reduced septal thickening and inflammatory cell infiltration. Altogether, these observations indicate that diHEPAs confer protection against LPS-induced ALI by restraining inflammatory cell infiltration and limiting neutrophil-associated tissue injury.

Exposure to LPS induces a robust inflammatory cascade marked by the excessive production of proinflammatory mediators, which play a pivotal role in the initiation and progression of acute and chronic inflammatory diseases [36]. In this study, pretreatment with diHEPAs markedly attenuated LPS-induced inflammatory responses in both RAW264.7 macrophages and mice with ALI, as evidenced by the significantly reduced secretion of TNF-α, IL-6, and IL-1β. In parallel, treatment with diHEPAs suppressed the production of NO and PGE2, two key inflammatory mediators primarily regulated by iNOS and COX-2, respectively [37]. Consistently, the expression levels of iNOS and COX-2 were substantially downregulated after treatment with diHEPAs, indicating the widespread inhibition of inflammatory effector pathways. Activation of the NF-κB signaling pathway is a key event in LPS-driven inflammation and contributes to the sustained expression of proinflammatory genes during inflammatory lung injury [38,39]. Our findings demonstrate that diHEPAs effectively mitigate inflammatory amplification at least partially through the NF-κB pathway. Notably, despite structural differences between 5,15-diHEPA and 8,15-diHEPA, their comparable bioactivity suggests functional convergence on shared signaling pathways, potentially involving overlapping GPCR-mediated mechanisms that have been described for SPMs [40].

Neutrophils are a major source of oxidative tissue injury in ALI, primarily through the release of MPO, a heme-containing enzyme abundantly stored in neutrophil azurophilic granules [41]. Upon neutrophil activation and degranulation, MPO is rapidly released into the extracellular space, where it uses hydrogen peroxide to generate highly reactive oxidants, such as hypochlorous acid. These oxidants directly damage alveolar epithelial and endothelial cells, thereby contributing to alveolar–capillary barrier dysfunction [33]. Our results revealed increased MPO activity in the lung tissue of mice with ALI, which was significantly suppressed by diHEPA treatment.

Oxidative stress is a critical pathogenic factor in ALI [42,43]. MPO-derived oxidants also amplify oxidative stress and promote lipid peroxidation. Excessive ROS production not only causes direct epithelial and endothelial injury but also reinforces proinflammatory macrophage activation [14]. In our study, treatment with diHEPAs markedly reduced the intracellular ROS levels and suppressed NOX2 activation. In parallel, increased Nrf2 nuclear accumulation and upregulation of Nrf2 target gene HO-1 were detected in RAW264.7 macrophages, consistent with the resolution-associated redox rebalancing after the suppression of inflammation. LPS-induced ALI is expected to be associated with excessive oxidative stress, as indicated by increased MDA levels and reduced antioxidant enzyme activity in lung tissues. Furthermore, treatment with diHEPAs normalized the redox balance, as evidenced by increased activities of SOD and decreased levels of MDA. Hence, our findings demonstrate that diHEPAs significantly suppress oxidative damage. From a translational perspective, these properties highlight their potential as endogenous, low-dose therapeutic candidates for ALI, offering advantages over conventional anti-inflammatory strategies that primarily target single pathways. However, given that our findings are largely based on pretreatment models, future studies are required to evaluate the therapeutic efficacy of diHEPAs when administered after disease onset, which would more closely mimic clinical scenarios [44]. These findings are in line with previous studies demonstrating that bioactive lipids, particularly SPMs such as resolvins and protectins, attenuate ALI by reducing neutrophil infiltration, suppressing pro-inflammatory cytokine production, and promoting resolution of inflammation [45,46]. In this context, our results extend the current understanding by identifying EPA-derived dihydroxylated metabolites as additional modulators within this lipid mediator network, suggesting that structurally distinct EPA derivatives can achieve functionally convergent protective effects in ALI.

## 4. Materials and Methods

### 4.1. Preparation of diHEPAs

**Figure 6:**
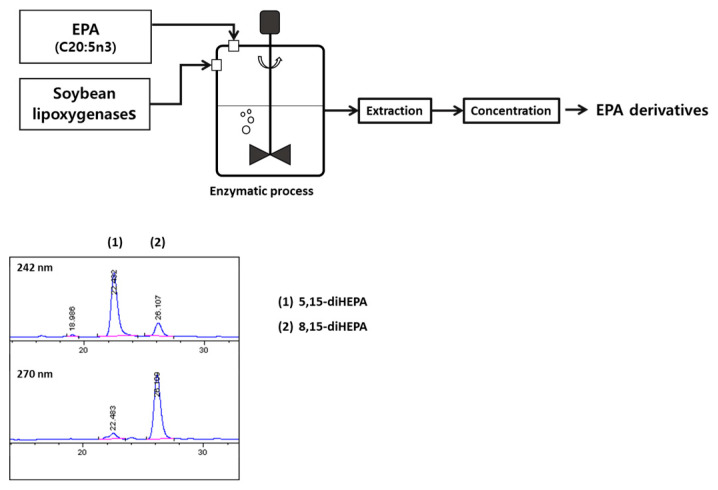
Preparation of diHEPAs by enzymatic reaction.

The formation of two dihydroxy derivatives, 5,15-HEPA and 8,15-diHEPA, from the polyunsaturated fatty acid EPA by the action of soybean lipoxygenase has previously been reported by Dobson et al. [32]. In the present study, in order to investigate for the cellular activity and the efficacy of oral administration in animal models of these two EPA hydroxyl derivatives, 5,15-HEPA and 8,15-diHEPA, an enzymatic reaction was carried out using lipoxygenase from whole aqueous extracts of soybean flour [47]. The enzymatic reaction of soybean lipoxygenase and the analysis of the reaction products were performed according to previously established methods in our laboratory [48]. Products bound to HP20 were eluted with ethanol and used for normal-phase high-performance liquid chromatography (NP-HPLC) analysis. NP-HPLC of diHEPAs was performed using a SUPELCOSIL LC-DIOL column (SUPELCO, Bellefonte, PA, USA, 25 × 3 mm, 5 μm). The mobile phase consisted of heptane/2-propanol/acetic acid (95:5:0.1, *v*/*v*/*v*) at a flow rate of 0.5 mL/min, with the column temperature maintained at 10 °C. diHEPAs were detected by monitoring UV absorbance at 242 and 270 nm. As a result, as shown in Figure 6, the formation of 5,15-HEPA and 8,15-diHEPA was confirmed. The catalyzed products were extracted using an HP20 resin.

### 4.2. Animals and Treatment

Female BALB/c mice (aged 8 weeks, weighing 20–25 g) were provided by Orient Bio. (Seongnam-si, Gyeonggi-do, Republic of Korea) and maintained under SPF conditions (22 ± 2 °C; 65 ± 5% humidity; 12-h light/dark cycle) with free access to food and water [49]. The experimental protocols were authorized by the Institutional Animal Care and Use Committee of the Korea Research Institute of Bioscience and Biotechnology as well as the Institutional Animal Ethics Committee (KRIBB-AEC-25428). Mice were randomly assigned to the following groups (*n* = 5/group): normal control (NC; saline), LPS, LPS + 5,15-diHEPA (40 μg/kg), LPS + 8,15-diHEPA (40 μg/kg), and LPS + combination treatment (5,15-diHEPA 20 μg/kg + 8,15-diHEPA 20 μg/kg). The dosing of diHEPAs was based on preliminary dose optimization studies and prior literature on bioactive lipid mediators. diHEPAs were orally administered for 7 days. At 1 h after the last final administration of diHEPAs, the mice were intranasally instilled with LPS (5 mg/kg, *Escherichia coli* O111:B4, Sigma, St. Louis, MO, USA) [41].

### 4.3. Collection and Analysis of BALF

At 24 h post-LPS administration, mice were euthanized, and BALF was collected by intratracheal infusion of 1 mL cold PBS. BALF samples were centrifuged at 400× *g* for 10 min at 4 °C. The levels of cytokines, including IL-6 (ab222503), TNF-α (ab208348), and IL-1β (ab100704), were measured using ELISA kits according to the manufacturer’s protocol (Abcam, Cambridge, UK).

### 4.4. Lung Wet-to-Dry Weight Ratio

Pulmonary edema was evaluated by measuring the lung wet-to-dry weight (W/D) ratio [50]. Lung tissue samples were weighed immediately after excision (wet weight) and then dried at 60 °C for 48 h to determine the dry weight.

### 4.5. Histopathological Analysis

Lung tissue samples were fixed in 4% paraformaldehyde (Bio-solution, Suwon city, Kyonggi-do, Republic of Korea), processed for paraffin embedding, and sliced into 5-μm sections. After hematoxylin and eosin staining (Abcam), histological changes were examined under a light microscope. All histological scoring was performed in a blinded manner to eliminate observer bias. Tissue damage was evaluated using a semi-quantitative histological scoring system based on established criteria. Specifically, the overall injury score was calculated as the sum of individual parameters, including alveolar wall thickening, inflammatory cell infiltration, edema formation, hemorrhage, each graded on a scale of 0–4 (0 = none, 1 = mild, 2 = moderate, 3 = marked, 4 = severe), for a total score ranging from 0–16 [51].

### 4.6. Cell Culture and Cell Viability Assay

RAW264.7 cells (KCLB-40071, mycoplasma-negative, Korea Cell Line Bank, Seoul, Republic of Korea) were cultured in DMEM (containing 10% FBS, 100 U/mL Penicillin, and 100 μg/mL streptomycin) in a 37 °C incubator with 5% CO_2_. Cells were seeded in 96-well plates at a density of 1 × 10^4^ cells per well (100 μL) and cotreated with diHEPA mixture (2, 4, 8, 20, 40, and 80 μg/mL) and LPS (1 μg/mL) for 24 h. Then, to each well, 100 μL of 3-(4,5-dimethylthiazol-2-yl)-2,5-diphenyl tetrazolium bromide (MTT, Abcam) solution was added and cultured for another 3 h. After discarding the medium in the well, 100 μL of dimethyl sulfoxide (DMSO, Sigma, St. Louis, MO, USA) was added to each well, and the plate was placed in the incubator for 10 min. Then, the absorbance of the well was measured at 570 nm using a microplate reader (Bio-Rad, Hercules, CA, USA).

### 4.7. Inflammation Assay In Vitro

Cells were seeded into 96-well plates at a density of 1 × 10^4^ cells per well (100 μL). The cells were treated with 5,15-diHEPA (8 μg/mL), 8,15-diHEPA (8 μg/mL), or a combination of 5,15-diHEPA (4 μg/mL) and 8,15-diHEPA (4 μg/mL) for 3 h and then explored to LPS (1 μg/mL). After 24 h of incubation, nitric oxide (NO) levels were measured using the Griess reagent (G2930; Abcam). The levels of IL-6 (ab222503), TNF-α (ab208348), IL-1β (ab100704), and PGE2 (MOEB2492; Assay Genie, Dublin, Ireland) in the supernatants were determined using ELISA kits.

### 4.8. Intracellular ROS Measurement

Cells were seeded into 24-well plates at a density of 4 × 10^4^ cells per well (500 μL) treated with diHEPAs and LPS for 24 h. Cells were exposed to DCFH-DA solution (Abcam) at 10 μM for 30 min. ROS levels were determined using fluorescence microscopy (Leica, Bensheim, Germany).

### 4.9. Evaluation of Antioxidant Activities

MPO levels in the lung tissue were determined using an ELISA kit (RAB0374; Millipore Sigma, Burlington, MA, USA). The levels of MDA (ab118970) and the activities of SOD (ab65354) in the lung tissue or cells were evaluated using a colorimetric assay kit (Abcam).

### 4.10. Quantitative Real-Time PCR

Total RNA was extracted from lung tissues or macrophages using TRIzol reagent. Quantitative real-time PCR (qRT-PCR) was performed using SYBR Green Master Mix on a real-time PCR system. Gene expression levels of iNOS and COX-2 were normalized to β-actin and calculated using the 2^−ΔΔCt^ method. The following primers were used: COX-2, forward: GCGACATACTCAAGCAGGAGCA, reverse: AGTGGTAACCGCTCAGGTGTTG; iNOS, forward: GAGACAGGGAAGTCTGAAGCAC, reverse: CCAGCAGTAGTTGCTCCTCTTC; β-actin, forward: CATTGCTGACAGGATGCAGAAGG, reverse: TGCTGGAAGGTGGACAGTGAGG.

### 4.11. Western Blot Analysis

Protein extracts were prepared from lung tissues or macrophages using RIPA buffer containing protease and phosphatase inhibitors. The total protein content was measured using a BCA kit (Thermo Fisher Scientific, Waltham, MA, USA). Equal amounts of protein were separated by SDS–PAGE and transferred to PVDF membranes. The membranes were incubated overnight at 4 °C with primary antibodies against NOX2 (ab310337), Nrf2 (ab92946), HO-1 (ab52947), p65 (ab16502), pp65 (ab76302), tubulin (ab52866), and GAPDH (ab181602) and then with HRP-conjugated goat anti-rabbit secondary antibody (ab205718) for 2 h at 37 °C. After washing the membranes with TBST (Bio-solution), signals were detected using an ECL substrate (Thermo Fisher Scientific) and visualized on film (Thermo Fisher Scientific). Band intensity was analyzed using ImageJ software (1.48v; National Institutes of Health).

### 4.12. Statistical Analysis

Data are expressed as mean ± standard deviation. Statistical analysis was conducted using one-way ANOVA followed by Tukey’s post hoc test for parametric data or the Kruskal–Wallis test followed by Dunn’s post hoc test for nonparametric data (used for injury score) using GraphPad Prism 9.5.1. *p* < 0.05 was considered to indicate a statistically significant difference.

## 5. Conclusions

diHEPAs exert protective effects against lung tissue damage in ALI by inhibiting inflammation and altering oxidative stress. The ability of diHEPAs to attenuate lung injury, reduce inflammatory cell infiltration, and preserve alveolar–capillary barrier integrity highlights their potential as low-dose, mechanism-based therapeutic agents. Furthermore, these findings suggest that enhancing the endogenous conversion of EPA to bioactive diHEPAs, either through dietary supplementation or enzymatic modulation, may represent a novel and clinically feasible strategy for controlling excessive inflammation in ALI/ARDS.

We acknowledge several limitations of this study. Although macrophage polarization and oxidative stress were the primary focus of our study, other immune and structural cell types, such as epithelial cells and endothelial cells, may also contribute to the diHEPA-mediated protection. Moreover, long-term outcomes, including fibrosis development and functional lung recovery, were beyond the scope of this study and require further investigation. Future studies should evaluate post-treatment paradigms, which are more directly translatable to clinical settings. In addition, this study was conducted exclusively in female mice; given known sex-dependent differences in immune and inflammatory responses, inclusion of male animals in future studies will be important to determine the generalizability of these findings.

## Data Availability

The original contributions presented in this study are included in the article. Further inquiries can be directed to the corresponding author.
